# Experiences, perceptions and potential impact of community‐based mentor mothers supporting pregnant and postpartum women with HIV in Kenya: a mixed‐methods study

**DOI:** 10.1002/jia2.25843

**Published:** 2021-11-19

**Authors:** Anna Helova, Maricianah Onono, Lisa L. Abuogi, Karen Hampanda, Kevin Owuor, Tobias Odwar, Sandhya Krishna, Gladys Odhiambo, Thomas Odeny, Janet M. Turan

**Affiliations:** ^1^ Department of Health Care Organization and Policy and Sparkman Center for Global Health, School of Public Health The University of Alabama at Birmingham Birmingham Alabama USA; ^2^ Centre for Microbiology Research Kenya Medical Research Institute Nairobi Kenya; ^3^ Department of Pediatrics, School of Medicine University of Colorado Denver Aurora Colorado USA; ^4^ Department of Obstetrics and Gynecology University of Colorado Denver, Anschutz Medical Campus Aurora Colorado USA; ^5^ Department of Biostatistics, School of Public Health The University of Alabama at Birmingham Birmingham Alabama USA; ^6^ Department of Medicine University of Missouri Kansas City Missouri USA

**Keywords:** adherence, Kenya, prevention of mother‐to‐child transmission of HIV, pregnant/postpartum women living with HIV, community‐based mentor mothers

## Abstract

**Introduction:**

Community‐based mentor mothers (cMMs) are women living with HIV who provide peer support to pregnant/postpartum women living with HIV (PWLWH) to enhance antiretroviral therapy (ART) adherence, retention in care and prevent perinatal transmission of HIV. The goal of this study was to explore the experiences, perceptions, mechanisms and health impact of cMMs on PWLWH in Kenya from the perspective of cMMs.

**Methods:**

We conducted a prospective mixed‐methods study in southwestern Kenya in 2015–2018. In the qualitative phase, we completed in‐depth interviews with cMMs to explore their perceptions and experiences in supporting PWLWH. Transcripts were broad‐coded according to identified themes, then fine‐coded using an inductive approach. In the quantitative phase, we analysed medical record data from PWLWH who were randomized in the cMM intervention to examine the impact of cMM visits on optimal prevention of mother‐to‐child transmission (PMTCT). We used cluster‐adjusted generalized estimating equation models to examine relationships with a composite outcome (facility delivery, infant HIV testing, ART adherence and undetectable viral load at 6 weeks postpartum). Finally, qualitative and quantitative results were integrated.

**Results:**

Convergence of findings from cMM interviews (*n* = 24) and PWLWH medical data (*n* = 589) revealed: (1) The cMM intervention was utilized and perceived as acceptable. PWLWH received, on average, 6.2 of 8 intended home visits through 6 weeks postpartum. (2) The cMMs reported serving as role models and confidantes, supporting PWLWH's acceptance of their HIV status, providing assurances about PMTCT and assisting with male partner disclosure and communication. cMMs also described benefits for themselves, including empowerment and increased income. (3) The cMM visits supported PWLWH's completion of PMTCT steps. Having ≥4 cMM home visits up to 6 weeks postpartum, as compared to <4 visits, was associated with higher likelihood of an optimal PMTCT composite outcome (adjusted relative risk 1.42, *p* = 0.044).

**Conclusions:**

We found that peer support from cMMs during pregnancy through 6 weeks postpartum was associated with improved uptake of critical PMTCT services and health behaviours and was perceived as beneficial for cMMs themselves. CMM support of PWLWH may be valuable for other low‐resource settings to improve engagement with lifelong ART and HIV services among PWLWH.

## 1 INTRODUCTION

In 2015, Kenya rolled out Option B+ [1, 2], a policy to implement universal, lifelong antiretroviral therapy (ART) for all pregnant and breastfeeding women living with HIV (WLWH), recommended by the World Health Organization (WHO) in settings with generalized HIV epidemics [3, 4]. By 2017, the perinatal transmission rate at 18 months of age had decreased from 14% in 2013 to 11.5%, and new paediatric HIV infections in children 0–14 years decreased by 38% in Kenya [5]. Lifelong ART in WLWH holds promise for improving maternal and child health, but critical challenges remain in achieving long‐term ART adherence and retention in HIV care due to individual, socio‐cultural and healthcare system challenges [5]. Persistent gaps, which are exacerbated by a lack of trained healthcare personnel, remain at each step of the prevention of mother‐to‐child transmission (PMTCT) continuum, including women who do not engage in HIV testing, link to and initiate ART treatment, maintain ART adherence or remain engaged in HIV care from pregnancy throughout postpartum [6].

To address ongoing challenges related to perinatal HIV prevention, WHO and the Joint United Nations Programme on HIV/AIDS (UNAIDS) recommend taskshifting, community involvement and engagement of WLWH in service provision [7, 8, 9, 10, 11]. Research shows that services provided by trained lay community health workers focusing on WLWH can promote adherence to important health behaviours through shared culture, language and community [12]. Additionally, lay community health workers have been shown to promote increased mother‐to‐child transmission (MTCT) awareness, retention, adherence to care and infant testing [13].

Mentor mothers (MMs) are a specific type of lay healthcare worker shown to increase the uptake of PMTCT services in several settings in sub‐Saharan Africa (SSA). The largest MM program, Mother2mothers, was established in South Africa in 2001 and has since been implemented across numerous settings in SSA, including Kenya [13, 14, 15, 16, 17, 18, 19, 20, 21]. MMs in such programs are WLWH who have been through PMTCT in the past 6 months to 2 years, are adhering well to treatment and have disclosed their HIV status. MMs provide one‐on‐one peer education to pregnant/postpartum women living with HIV (PWLWH) on PMTCT and health‐related topics, psychosocial support [17, 22, 23, 24, 25] and encouragement for ART adherence and retention in HIV care [23, 26]. MM programs have been promoted as an innovative and cost‐effective means to increase community employment and the availability of health services in underserved communities [27].

In Kenya and other settings, MMs have generally been based at health facilities, providing services to women when they come in to the clinic, as opposed to being implemented at the community level. In the Mother Infant Visit Adherence and Treatment Engagement (MOTIVATE) study [28], community‐based mentor mothers (cMMs) were recruited and trained to conduct community outreach and home visits for PWLWH to promote maternal and child health as well as PMTCT service uptake [29]. The goal of this study was to explore the experiences, perceptions, mechanisms and health impact of cMMs on PWLWH in Kenya from the perspective of cMMs.

## 2 METHODS

This study was conducted in the context of a larger parent trial, The MOTIVATE study, that utilized a 2 × 2 cluster‐randomized factorial research design to examine the individual and combined effects of two interventions (cMMs and text messaging) on PWLWH ART adherence and retention in care compared to standard care. MOTIVATE study participants were pregnant WLWH 18 years and older accessing care at one of 24 government antenatal clinics randomized into one of the four study arms in Homa Bay, Migori and Kisumu counties of southwestern Kenya; three of the most highly HIV‐affected counties in Kenya [30, 31]. Study participants were enrolled during pregnancy from December 2015 to August 2017 and followed until at least 12 months postpartum. More details on the parent study are published elsewhere [28]. In the MOTIVATE study, cMMs were selected based on results from formative research that explored “ideal” characteristics of cMMs based on perspectives from PWLWH, their male partners and healthcare workers. The results of this formative research have been published elsewhere [29]. Based on this study and previous experiences in this setting, cMMs did not wear uniforms or use vehicles with any distinguishing branding related to HIV services. CMMs were trained and followed a detailed protocol of 13 home visits during pregnancy/postpartum (four during pregnancy, four in early postpartum and five from 6 weeks up to 12 months after the birth). Fidelity was maintained via bimonthly observed visits by study coordinators using a standardized evaluation tool, monitoring of visit logs and monthly data review [32]. If necessary, these assessments were conducted more frequently. CMMs received a small fixed monthly stipend similar to that received by facility‐based MMs, a standard position at clinics in Kenya. Separate analyses presented elsewhere have examined outcomes up to 12 months postpartum for the entire trial. Time‐to‐event analyses revealed that women who received the cMM and text arm intervention, particularly those who received a higher dose of the intervention (at least 80%), were more likely to be retained in care at 12 months after the birth compared to those randomized into a standard of care. However, adherence and viral load (VL) suppression at 12 months postpartum did not differ by study arm [33].

### 2.1 Study design

In this sub‐study, we aimed to understand how cMMs influence PWLWH's health behaviours and outcomes in Kenya, utilizing a prospective, convergent parallel mixed‐methods study design, integrating qualitative and quantitative findings [34].

### 2.2 Qualitative phase

We conducted 24 individual in‐depth interviews with MOTIVATE study cMMs from 10 study communities between Apriland May 2016 to explore the perceptions of cMMs on their role in supporting PWLWH. CMM participant characteristics were collected, including demographics, pregnancy and HIV‐related information. Interviews were conducted in private settings in English or Dhuluo, by one of the two women interviewers. A qualitative in‐depth interview guide was developed based on a review of the literature, prior studies on pregnancy and HIV in this setting [35, 36] and preliminary results from the formative phase of the parent MOTIVATE study [28]. The main topics explored included: (1) experience working as a cMM (responsibilities, benefits/challenges and perceived impact of their work), (2) acceptability of cMMs in the community, (3) impact of this work on cMMs themselves and (4) suggestions for improving the cMM intervention.

Interviews were digitally recorded, translated to English if applicable and transcribed verbatim, excluding any identifying information. Subsequently, transcripts were coded by two researchers (AH and SK) using the Dedoose qualitative software, and consistency of coding between two individuals was established by initially double‐coding the same transcripts and, after establishing good agreement on codes and meanings, through frequent discussion and validation between coders and the wider team of investigators. Coding and analysis followed a thematic analysis approach [37, 38]. The coding framework was developed based on the literature, topics from interview guides and emerging themes from transcripts. Transcripts were initially broad‐coded, then fine‐coded using an inductive approach. Major themes were refined and sub‐themes identified.

### 2.3 Quantitative phase

We performed a secondary analysis of prospective quantitative data from the MOTIVATE study to understand how cMMs’ support impacted the uptake of PMTCT services and health behaviours among all retained 589 PWLWH (88.7%) aged ≥18 years randomized to one of the cMM intervention arms of the trial. Women not retained were lost‐to‐follow‐up, discontinued from the study, died or experienced pregnancy/child loss. Data on study indicators and socio‐demographic characteristics were abstracted from medical records by study data clerks for the period from study enrolment in pregnancy up to 6 weeks postpartum, a particularly vulnerable period for mothers and infants.

#### 2.3.1 Outcomes and predictors

We explored predictors of two binary primary outcomes and one binary composite outcome: (1) self‐reported good (>95% ART adherence at 6 weeks postpartum extracted from medical records vs. ≤95%); (2) detectable versus undetectable (below the lower test limit) VL at 6 weeks postpartum and viral suppression was defined as VL between 0 and 1000 copies/ml; and (3) an achievement of all components of an optimal PMTCT composite outcome, consisting of critical steps of perinatal transmission of HIV (mother's >95% ART adherence and undetectable VL at 6 weeks postpartum, health facility delivery and infant HIV testing at 6 weeks after birth), versus women who did not achieve all four components. Exclusive breastfeeding was excluded from the composite variable due to very high reported rates across the sample, with almost no variability.

The primary predictor was the total number of cMM visits that PWLWH received during pregnancy and up to 6 weeks postpartum, categorized as a binary variable into ≥4 versus <4 cMM visits. The cut‐off represented half of visits recommended for this period. Other independent variables included in these analyses as potential predictors of PMTCT outcomes based on the literature, included baseline socio‐demographic and patient characteristics: age (categorized into <25 vs. ≥25 years), marital status (married vs. not), gravida (primigravid vs. ≥2 pregnancies), gestational age at the first antenatal visit during this pregnancy (≤28 vs. >28 weeks) [39, 40, 41], study arm (cMM vs. cMM+text messages), timing of HIV diagnosis (women newly diagnosed with HIV at their first antenatal visit of this pregnancy vs. known HIV‐positive prior to the current pregnancy) [39], male partner's baseline HIV status (positive vs. negative/unknown status) and disclosure to a male partner by the time of the birth [42, 43, 44].

#### 2.3.2 Quantitative data analyses

All statistical analyses were conducted using Stata SE 16. Distributions of all variables were examined with descriptive statistics, counts (%) and means (± standard deviations) [45]. Missing data were examined and sensitivity analyses were performed to investigate the effects of missing data on primary outcomes. There was no significant effect of missing data on the outcomes. Variables were tested for multicollinearity, and strongly correlated variables (*r* ≥0.60) were excluded from the model. Covariates with a *p*‐value of <0.2 in univariable analyses were included in multivariable models. Given the clustered study design of the MOTIVATE study (24 sites), generalized estimating equation models were used to test for differences of interest in univariable and multivariable models, adjusted for relevant covariates and accounting for site‐level clustering using robust variance estimates. Results were summarized as relative risks for models with binary response outcomes. Relative risk estimates were selected instead of the odds ratios as a more accurate measure for prospective data, and given the high prevalence of the outcome measures. Statistical significance was set at 5% level (*p*‐value <0.05). The analyses were adjusted for weeks of pregnancy at the start of the study, given that this variable influenced the duration of time that women had available to receive the stipulated number of cMM visits.

### 2.4 Integration of qualitative and quantitative findings

At the interpretation stage, we made comparisons of the findings from the qualitative and quantitative data. We first prepared separate reports on qualitative and quantitative findings. Then, for each thematic area, we examined both the qualitative and quantitative data using joint displays to elucidate the findings that emerged from each method and to identify areas where the findings converged, diverged or added insight to one another [46]. This approach provided a rich set of conclusions, and helped to triangulate findings that may have been difficult to interpret using either method on its own [47]. For interpretation, we placed equal weight on the qualitative and quantitative findings [48].

### 2.5 Ethical approvals

Ethical approvals were obtained from the University of Colorado Denver, University of Alabama at Birmingham, and Scientific and Ethics Review Unit in Kenya. All study participants provided written informed consent. Clear explanations were given to PWLWH and cMMs that the study participation was voluntary and separate from their employment/medical care.

## 3 RESULTS

### 3.1 Qualitative findings

All 24 study cMMs (mean age 34.2±6.7 years) agreed to participate in the individual interviews. The majority of cMMs were married (62.5%), living in an HIV‐positive concordant relationship (58.3%) and had, on average, 2.9 living children. All cMMs completed at least primary education, and 62.5% completed secondary/higher education. Socio‐demographic and HIV‐related characteristics of participants are presented in Table 1.

**Table 1:** Socio‐demographic and HIV‐related characteristics of study participants

Characteristics	Community mentor mothers (*n*=24)	Pregnant women living with HIV (*n*=589)
Variables reported at study baseline:		
Age (mean, SD)	34.2 (±6.7)	28.9±5.6
Age category *n* (%)		
<25 years		169 (28.7%)
≥25 years		419 (71.3%)
Education *n* (%)		
Did not complete secondary	9 (37.5%)	Not available
Completed secondary educ.	15 (62.5%)	
Number of living children (cMMs)/number of pregnancies (pregnant women) (mean, SD)	2.9 (±1.3)	3.5 (±1.7)
1		43 (7.3%)
≥ 2		545 (92.7%)
Weeks of pregnancy when started study *n* (%)	N.A.	23.6 (±7.6)
≤ 28 weeks gestational age		439 (74.7%)
> 28 weeks gestational age		149 (25.3%)
Marital status *n* (%)		
Married	15 (62.5%)	545 (92.7%)
Not married	9 (37.5%)	43 (7.3%)
HIV at first antenatal visit *n* (%)		
Newly positive	N.A.	101 (17.2%)
Known positive		487 (82.8%)
Partner's HIV status *n* (%)		
Positive	14 (58.3%)	108 (18.6%)
Negative	8 (33.3%)	23 (4.0%)
Unknown	2 (8.3%)	451 (77.5%)
Intervention arm *n* (%)	N.A.	
cMM		297 (50.5%)
cMM + text messaging		291 (49.5%)
Variables captured after baseline:		
Total # cMM total visits (mean, SD)	N.A.	6.2 (±1.9)
Visit categories *n* (%)		
0		7 (1.2%)
1		11 (1.9%)
2		13 (2.2%)
3		24 (4.1%)
4		39 (6.7%)
5		80 (13.8%)
6		108 (18.6%)
7		108 (18.6%)
8		192 (33.0%)
Disclosure to male partner at baseline *n* (%)	N.A.	
Yes		545 (92.7%)
No		43 (7.3%)
Disclosure to male partner at the time of birth	N.A.	
*n* (%)		
Yes		553 (96.3%)
No		21 (3.7%)
Infant exclusive breastfeeding at 6 weeks after birth *n* (%)	N.A.	
Yes		*N*=493
No		492 (99.8%)
Missing		1 (0.2%)
Mother's ART adherence at 6 weeks postpartum *n* (%)	N.A.	
Good (>95%)		564 (97.1%)
Less than good (≤95%)		17 (2.9%)
Mother's VL at 6 weeks postpartum *n* (%)	N.A.	*N*=408
Undetectable (0 copies/ml)		295 (72.3%)
Viral suppression (>0, <1000 copies/ml)		88 (21.6%)
Not virally suppressed (1000 copies/ml)		25 (6.1%)
Health facility delivery *n* (%)	N.A.	
Yes		538 (91.5%)
No		50 (8.5%)
Infant testing at 6 weeks after birth *n* (%)	N.A.	
Yes		570 (97.9%)
No		12 (2.1%)
Optimal PMTCT composite outcome^a^ *n* (%)	N.A.	*N*=374
Yes		261 (69.8%)
No		80 (30.2%)

Abbreviations: ART, antiretroviral therapy; cMM, community‐based mentor mothers; PMTCT, prevention of mother‐to‐child transmission; SD, standard deviation; VL, viral load.

^a^ Optimal composite outcome (infant testing at 6 weeks, facility delivery, good adherence and undetectable VL at 6 weeks postpartum).

Five major themes were identified through the thematic analysis of how cMMs perceive their role in supporting PWLWH.

#### 3.1.1 Theme 1: High perceived acceptability of the cMM intervention by PWLWH and community

Most of cMMs believed that the cMM intervention is well accepted by PWLWH due to their provision of services that are private and convenient with dedicated individual attention.

*“As the people doing the visits, they know us, they take us as sisters [nurses] who they can share their problems with. The moment you go to them in their houses, there they are so free and confide in you whenever they face problems.”* (31 years, 3 children)

Despite risks of inadvertent disclosure of their own HIV status, cMMs preferred working in their own community due to their familiarity with community culture and beliefs, and close geographical proximity to the PWLWH served, particularly during births and emergency situations.

*“It is good because in the community where you live you will know the behavior of people. You will also know the kind of problems women have. You know when you are close to her, she will come and tell you her problems in a better way compared to those from far.”* (28 years, 2 children)

#### 3.1.2 Theme 2: cMM roles

cMMs described taking pride in being “on‐call” around the clock and going beyond their formal duties. They felt they are serving as role models and confidants to PWLWH, helping with acceptance of HIV‐positive status, providing encouragement about the potential of having an HIV‐negative child and assisting with partner disclosure and communication.

*“I wanted them to know that you can give birth and raise a HIV negative baby. It was something I had experienced, and did as I was taught and I was successful. You know, when you are teaching someone something that you have experienced, it becomes very easy, and the person also understands quickly*.” (41 years, 4 children)

cMMs described how they link and refer women to HIV care, PMTCT, and maternal and child health services, and provide tangible support, including the development of birth plans, medications pick‐up and even planning of household finances.

*“What makes me happy is that they listen to what I teach them, I tell them to take a motorbike to the hospital whenever they are in labor. I advise them to save some money to get to the hospital, and that's what they've been doing.”* (42 years, 1 child)

#### 3.1.3 Theme 3: Positive impact of cMM work on PWLWH and their families

All interviewed cMMs expressed pride and believed in the value of their work, particularly in contributing to improved ART adherence and retention in HIV care, decisions to deliver at a health facility, increased infant HIV testing/adherence to ARV, infant immunizations and safe infant feeding.

*“The hospital deliveries have increased, that one I know. […] When we sit down with them, we go through the pros and cons of home delivery, we compare the two. […] We keep on reminding them about drug adherence. You find a mother who is stressed up.[…] She has forgotten everything. By going there, at least some adherence is maintained.” (51 years, 1 child)*

Many cMMs mentioned increased rates of HIV‐status disclosure among their clients, and felt that improved couple communication and male involvement were some of the most critical impacts of their work.

*“For those who have not disclosed to their husbands, we teach them how to disclose or we ask them to bring their partners for couple testing. This has built friendship between us. Those who used to disappear after testing positive and picking their drugs for two weeks, nowadays do not disappear.”* (41 years, 4 children)

#### 3.1.4 Theme 4: Challenges of cMM work

CMMs expressed challenges faced when visiting PWLWHs’ homes. Women's initial nondisclosure of HIV status, especially to a male partner but also other household/community members, was mentioned as a significant challenge, particularly immediately after the birth. According to cMMs, nondisclosure led to lack of male partner involvement in the pregnancy and support for PMTCT step completion. The cMMs explained that disclosure to a male partner was avoided by women due to their fears of being blamed for bringing HIV into the relationship, physical abuse and possibly being forced to leave the home. The context of disclosure became even more complex in polygamous marriages, a common relationship type in this area. CMMs emphasized that even women who have not disclosed their status want to participate in cMM visits; however, a cMM must be mindful of potential inadvertent disclose of the woman's HIV status.

*“You see, these people who have not disclosed can accept that you go and visit them at home. Then the husband catches you abruptly. […] That may bring a quarrel. They know that people from the facility normally follow those on ARVs. […] When you are caught, you have to change the topic, then you give her another appointment. But you will not know what will happen after you have left.”* (41 years, 4 children)

Additionally, cMMs voiced concern about the ability to deliver three home visits during the first week after baby's birth, particularly if a woman has not disclosed her HIV status. CMMs also described logistical challenges when making home visits, some due to natural causes (rainy season, floods and mud), but also limited financial resources (insufficient cell phone minutes and lack of finances for transport). CMMs ultimately felt the necessity to visit their “clients” no matter what the circumstances.

*“Currently it's a rainy season. It rains heavily to an extent this place gets flooded. So sometimes you are so tired but then you have to go, and the place is muddy. […] Also, we travel long distances and this place is hilly. And you have to go […] to these people. You can't abandon them.”* (31 years, 3 children)

#### 3.1.5 Theme 5: Impact of being a cMM on self

Many cMMs expressed that being a cMM resulted in increased self‐worth, self‐empowerment and motivation to adhere to their own ART and HIV care.

*“*It *has really changed me […]because you feel so nice when a baby comes out negative. And it also feels good when you talk to a client. You tell her your experience, then she sees you as a friend.”* (24 years, 1 child)

Many cMMs expressed that their work experience as a cMM encouraged them to further their skills and training, possibly by becoming a nurse. CMMs also discussed that PLHIV are often stigmatized in the job market and that this study provided a unique opportunity for WLWH actually requiring an HIV‐positive status. Lastly, employment increased their income and provided them with much‐needed resources for themselves and their families.

*“*Being *a community mentor mother has helped me in so many ways, the people who looked down upon me now view me as a person of value. One that can impart some knowledge on them. My children are now able to go to school. But before, they wouldn't go for two weeks without being sent back home for fees[…]. Also, right now am able to dress neatly, am more knowledgeable about things.”* (28 years, 3 children)

### 3.2 Mechanisms of cMM influence on PMTCT steps completion

**Figure 1:**
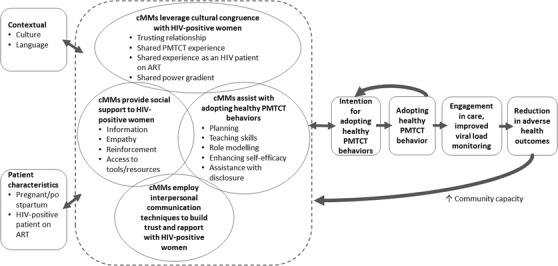
Adapted conceptual framework of community mentor mothers (cMMs) and PWLWH served, building on a Katigbak conceptual framework for the role of community health workers in facilitating patients’ adoption of health behaviours [12]. Abbreviations: ART, antiretroviral therapy; cMM, community‐based mentor mothers; PMTCT, prevention of mother‐to‐child transmission.

The qualitative findings were used to adapt a conceptual framework of cMM's role in facilitating the completion of PMTCT steps (Figure 1), building on an existing conceptual framework for the role of community health workers on patients’ adoption of health behaviours [12]. CMMs provided social support, leveraged cultural congruence, shared PMTCT and ART experience, and had good communication with PWLWH, which led to building a trusting relationship and rapport to assist PWLWH with completing PMTCT steps. CMMs believed that one of the most important pathways to good PMTCT outcomes was through HIV status disclosure, particularly to a male partner. Good PMTCT behaviours were believed to result in an increased engagement in care, improved VL monitoring and a reduction of adverse health and PMTCT outcomes. The cMMs impacted not only women's intention to adopt healthy PMTCT behaviours, but also the actual adoption of these behaviours directly through provision of direct services, for example providing linkages and referrals to services, and helping with accessing those services. CMMs also described how the intervention increased community healthcare capacity by training community‐based WLWH as cMMs to work in the health field, and had positive effects on cMMs themselves.

### 3.3 Quantitative findings

Of the 589 PWLWH who received the cMM intervention (mean age 28.9±5.6 years), most were married (92.7%) and had ≥2 children (92.7%). The majority of women were enrolled in the MOTIVATE study prior to reaching 28 weeks of gestation (mean 23.6±7.6 weeks) and initiated ART prior to the index pregnancy (82.8%). According to medical records, most women did not know their male partner's HIV status at study enrolment (77.5%), and 18.6% lived in an HIV‐positive concordant relationship. Socio‐demographic and HIV‐related characteristics of participants are presented in Table 1.

The majority of women reported that they had disclosed their HIV status to their male partners by the time of delivery (96.3%). Most women (97.1%) self‐reported good adherence (>95% of the time) at 6 weeks postpartum clinical visit. About 72.3% of women achieved undetectable VL at 6 weeks postpartum, an additional 21.6% achieved viral suppression and 6.1% were not virally suppressed. Most women delivered their baby at a healthcare facility (91.5%), and 97.9% of the infants were tested for HIV at 6 weeks. The majority (99.8%) of women self‐reported exclusive breastfeeding at the 6 weeks postpartum clinical visit. Overall, nearly 70.0% of women (69.8%) achieved all composite PMTCT steps, consisting of good ART adherence and undetectable VL at 6 weeks postpartum, facility delivery and infant testing at 6 weeks. About one‐third of participants (33.0%) received the full dose of eight cMM visits during pregnancy and up to 6 weeks postpartum (mean 6.2±1.9 visits) and 5.3% received a very low *“*dose” of this intervention (≤2 cMM visits).

In univariable analyses, PWLWH who received more than four cMM visits during the period of pregnancy and up to 6 weeks postpartum were more likely to achieve the optimal PMTCT composite outcome [72.6% and 49.6%, respectively; relative risk (RR) = 1.09, *p*‐value = 0.034] (Table 2). This relationship persisted in the multivariable analysis after adjusting for covariates (adjusted RR = 1.42, *p*‐value = 0.044) (Table 3).

**Table 2:** Univariable analyses of the association between PWLWH characteristics and uptake of select PMTCT services and health behaviours

Factor	Mother's ART adherence at 6 weeks postpartum		Mother's undetectable VL at 6 weeks postpartum		Optimal PMTCT composite outcome	
	RR (95% CI)	*p*‐value	RR (95% CI)	*p*‐value	RR (95% CI)	*p*‐value
# of total cMM visits						
≤4	Ref.		Ref.		Ref.	
>4	1.02 (0.99–1.06)	0.200	1.35 (0.95–1.90)	0.091	1.46 (1.03–2.06)	0.034
Age at baseline						
<25 years	Ref.		Ref.		Ref.	
≥25 years	1.02 (0.98–1.06)	0.298	1.18 (0.98–1.41)	0.081	0.15 (0.97–1.36)	0.111
Gravida						
Primigravid	Ref.		Ref.		Ref.	
≥ 2	–	–	0.97 (0.77–1.23)	0.793	0.94 (0.74–1.19)	0.607
Weeks of pregnancy when started study						
≤ 28 weeks	Ref.		Ref.		Ref.	
> 28 weeks	1.01 (0.98–1.03)	0.670	0.93 (0.78–1.12)	0.470	0.94 (0.78–1.13)	0.495
Marital status						
Not married	Ref.		Ref.		Ref.	
Married	1.01 (0.96–1.06)	0.700	1.00 (0.87–1.15)	0.994	0.96 (0.82–1.12)	0.615
HIV at first ANC visit						
Newly positive	0.97 (0.94–1.00)	0.068	0.97 (0.76–1.23)	0.776	0.98 (0.76–1.26)	0.844
Known positive	Ref.		Ref.		Ref.	
Partner's HIV status						
Positive	Ref.		Ref.		Ref.	
Negative/unknown	1.00 (0.96–1.05)	0.919	1.04 (0.88–1.23)	0.616	1.04 (0.88–1.22)	0.658
Intervention arm						
Cmm	Ref.		Ref.		Ref.	
cMM + text messaging	1.01 (0.96–1.06)	0.658	0.96 (0.75–1.23)	0.757	0.99 (0.76–1.28)	0.912
Disclosure to male partner by the birth						
Yes	1.03 (0.92–1.16)	0.581	1.23 (0.69–2.17)	0.485	1.26 (0.64–2.49)	0.510
No	Ref.		Ref.		Ref.	

Abbreviations: ANC, antenatal visit; ART, antiretroviral therapy; CI, confidence interval; cMM, community‐based mentor mothers; PMTCT, prevention of mother‐to‐child transmission; PWLWH, pregnant/postpartum women living with HIV; VL, viral load.

**Table 3:** Multivariable analyses of the association between PWLWH characteristics and uptake of select PMTCT services and health behaviours

Factor	Mother's ART adherence at 6 weeks postpartum		Mother's undetectable VL at 6 weeks postpartum		Optimal PMTCT composite outcome	
	aRR^a^ (95% CI)	*p*‐value	aRR (95% CI)	*p*‐value	aRR (95% CI)	*p*‐value
# of total cMM visits						0.044
≤4	Ref.	0.177	Ref.	0.119	Ref.	
>4	1.02 (0.99–1.06)		1.31 (0.93–1.83)		1.42 (1.01–2.01)	
Age at baseline						0.093
<25 years	Ref.	0.245	Ref.	0.056	Ref.	
≥25 years	1.02 (0.98–1.07)		1.18 (1.00–1.39)		1.14 (0.98–1.33)	
Weeks of pregnancy when started study						
≤ 28 weeks	Ref.	0.108	Ref.	0.428	Ref.	0.511
> 28 weeks	1.02 (1.00–1.03)		0.93 (0.77–1.12)		0.94 (0.79–1.13)	
Partner's HIV status						0.316
Positive	Ref.	0.798	Ref.	0.262	Ref.	
Negative/unknown	1.00 (0.97–1.04)		1.11 (0.92–1.33)		1.10 (0.92–1.31)	

Abbreviations: aRR, adjusted relative risk; ART, antiretroviral therapy; CI, confidence interval; cMM, community‐based mentor mothers; PMTCT, prevention of mother‐to‐child transmission; PWLWH, pregnant/postpartum women living with HIV; VL, viral load.

^a^ The outcomes were adjusted for weeks of pregnancy at the start of the study, given that this variable influenced the duration of time that women had available to receive the stipulated number of cMM visits.

### 3.4 Integration of qualitative and quantitative results

Based on converged data, we identified the following key findings related to the impact of cMMs in supporting PWLWH, presented in Table 4: (1) The cMM intervention was utilized and perceived as acceptable based on the cMM perspectives and the number of home visits completed. (2) The cMMs described their main roles as supporting PWLWH's acceptance of their HIV status, providing assurances about PMTCT and assisting with male partner disclosure and communication. CMMs also reported positive impact on themselves, including empowerment and additional income. (3) CMM visits and their activities improved the completion of PMTCT steps by PWLWH. (4) Disclosure of HIV status to a male partner might be a contributing factor in improved PMTCT step completion. While qualitative and quantitative data summarized in themes 1–3 in Table 4 converged, the disclosure of HIV status and the mechanism of the impact of such disclosure through cMM assistance on improved PMTCT outcomes (theme 4 in Table 4) diverged and was not detected in our quantitative analysis.

**Table 4:** Side‐by‐side comparison of qualitative and quantitative results showing the impact of cMMs in supporting PWLWH in completion of PMTCT continuum steps

	Themes	In‐depth interviews with cMMs (*N* = 24)	Quantitative data (*N* = 589 PWLWH)
1.	Utilization and acceptability of the cMM intervention	cMMs perceived that their home visits were highly acceptable: Serve as role models and confidantes May be referred over the facility‐based mentor mothers due to privacy, convenience and dedicated attention	The overall number of cMM visits achieved may indicate high acceptability of the cMM visits. Participants had, on average, 6.2 out of 8 cMM visits during pregnancy and up to 6 weeks postpartum.
2.	CMM roles	Supporting acceptance of HIV status and providing encouragement about the potential of having an HIV‐negative child Assisting with partner disclosure/communication Linking and referring women to HIV care, PMTCT and maternal and child health services Providing tangible support (development of birth plans, medication pick‐up and help with household finances)	
3.	Improved PMTCT behaviours	CMMs reported that their clients achieved: Improved ART adherence and retention in care Delivery at a health facility Infant HIV testing, infant adherence to ARV, immunization and safe infant feeding	88.7% of women were retained in in the study and HIV care up to 6 weeks postpartum. Having four or more cMM visits in the peripartum period was associated with achieving optimal completion of PMTCT steps, including infant testing at 6 weeks, facility delivery, good ART adherence and undetectable VL at 6 weeks postpartum (aRR = 1.42, *p* = 0.044).
4.	Disclosure as a potential mechanism for the impact of cMM visits on outcomes	cMMs felt that one of the most important ways that their visits positively impacted outcomes was through assistance with male partner communication and disclosure	96.30% of women reported having disclosed their HIV status to their male partner by the time of the delivery. Disclosure of HIV status to a male partner by the time of the birth was not significantly associated with optimal PMTCT composite outcome (RR = 1.26, *p* = 0.510), mother's adherence at 6 weeks postpartum (RR = 1.03, *p* = 0.581) or maternal undetectable viral load at 6 weeks postpartum (RR = 1.23, *p* = 0.485).

Abbreviations: ART, antiretroviral therapy; cMM, community‐based mentor mothers; PMTCT, prevention of mother‐to‐child transmission; PWLWH, pregnant/postpartum women living with HIV; RR, relative risk; VL, viral load.

## 4 DISCUSSION

In this mixed‐methods study, we explored the experiences, perceptions and mechanisms through which cMMs provide support for PWLWH in an area of high HIV burden in southwestern Kenya. We found that the cMMs perceived this intervention as acceptable and effective in this setting. CMMs believed that their support to PWLWH during home visits improved maternal and infant outcomes through the relationships and trust they were able to establish with PWLWH. Further, the number of cMM home visits received by PWLWH was associated with improved completion of critical PMTCT steps.

These results are consistent with current research and practice indicating that the involvement of peer mentors living with HIV to support PMTCT services has multiple positive benefits for PWLWH and their infants, including initiation of pregnant women on ART [49, 50], uptake of PMTCT services and improved retention in care [18, 22, 51, 52, 53], as well as other PMTCT behaviours [9, 29, 54, 55]. Services provided by lay health workers have been also linked to increased facility deliveries [56], timely infant diagnosis [14, 15, 18, 49, 51, 57, 58] and exclusive breastfeeding [57, 59, 60]. However, PWLWH community peer support strategies have had mixed results when it comes to viral suppression of PWLWH [16, 61] and lower perinatal transmission [62]. CMMs in their interviews often voiced concern about the early postpartum period, and their ability to deliver the intervention and home visits to the full extent. We were not able to establish an individual impact of cMM visits on ART adherence or viral suppression at 6 weeks, but demonstrated improved suboutcomes within a composite PMTCT outcome.

Despite many benefits of taskshifting to lay health workers delivering services in the community [63], many have raised concerns about attrition rates of PWLWH [22, 61, 64], particularly in the postpartum period [65], as well as a lack of trust in community workers and lack of fidelity to the intended intervention [14, 15, 66, 67]. Other studies warn that the low remuneration of community health workers [68] and heavy workload [69] might undermine the delivery of such interventions. The cMMs interviewed in this study also perceived limited funding for their communication and transportation needs, the intensity of the intervention and unpredictable nature of their schedule as major challenges to the success of this intervention.

Many have also brought attention to potential negative impacts on peer mentors themselves, particularly inadvertent disclosure of their own status and stigma, and by extension inadvertent disclosure of clients. Concerns over confidentiality and status disclosure to a male partner and community while delivering community intervention have been well‐documented [32]. Numerous programs do not have community workers wear a uniform indicating that they are from the HIV clinic, in order to protect their community workers and clients served [50, 68]. Despite these concerns, home visits were feasible and acceptable in our study and cMMs demonstrated the ability to overcome these concerns.

Beyond the impact on PWLWH, we found a positive impact on cMMs themselves who described increased commitment to their own ART adherence and retention in care, and self‐empowerment. Other studies show how PWLWH perceive reduced stigma related to their HIV status based on the openness and acceptance of HIV status by their peer mentors [51].

Although cMMs described assisting with HIV status disclosure to male partners and felt it was one of the main mechanisms for the success of their work, we were not able to find evidence of this in our quantitative analysis. Existing evidence suggests that disclosure often positively affects uptake and retention in care for PWLWH [42, 43, 44]. Building on our own work [70] as well as several other reports from Kenyan and other Southern African sites, we speculate that HIV status disclosure to a male partner is complex and ongoing process, and might have bidirectional effects. While for some women and their infants, this disclosure results in a positive outcome, social support, adherence, reduced stigma and decreased MTCT [71, 72], for some, it might result in conflict, blame or intimate partner violence, particularly in complex relationships, for example polygamous marriages [70, 73, 74]. Additionally, our data on male partner disclosure were based on the self‐reported disclosure to a male partner to the clinician at clinic visits and showed a high level of disclosure compared to other community‐based surveys and studies conducted in this area [51, 75, 76, 77]. It is possible that true disclosure levels are lower than those reported at the antenatal clinics, due to social desirability bias. It is also possible that even low doses of the cMM intervention (<4 visits) were enough to support very high levels of male partner disclosure by the time that the women gave birth.

The robustness and potential applicability of these results to similar settings across SSA are strengthened by mixed‐methods study design, a large sample size consisting of both women on ART prior to the index pregnancy and newly diagnosed PWLWH, recruitment from 24 clinics in three counties in southwestern Kenya and a longitudinally observed cohort during pregnancy and up to 6 weeks postpartum. However, the study results should be considered within the context of some limitations and possible biases. In the qualitative phase, the results are based solely on the cMMs’ perceptions of the intervention. It is possible that PWLWH have different perspectives on the cMM intervention that were not conveyed by the cMMs, and there may have been some advantages or disadvantages of this intervention that we were not able to capture in the study. In the quantitative phase, the study recruited pregnant women visiting antenatal clinics who meet additional eligibility criteria (≥18 years, access to a mobile phone, and have disclosed their HIV status to any person sharing the phone and willing to have home visits). Thus, women enrolled might be different from women who did not meet these eligibility criteria. We analysed data for PWLWH in the cMM intervention arm regardless of if they received the full intervention; however, we excluded women not retained in the parent trial due to the loss‐to‐follow‐up, discontinuation from the study, death or pregnancy/child loss. Quantitative data were abstracted from medical records that have missing values, such as VL, and lack detailed socio‐demographics, social and psychosocial variables that are known to play an important role in PMTCT outcomes and adherence to care, for example socio‐economic status, mental health issues and intimate partner violence. Some information in the medical records, such as disclosure to the male partner or self‐reported ART adherence, are subject to strong recall and social desirability bias as they are reported to healthcare providers who may scold them for reporting sub‐optimal behaviours. Additionally, the study was conducted in the context of frequent changes to policies and standard care provision, as well as service disruptions due to adverse weather conditions and health worker strikes. However, the roles and importance of lay/peer healthcare workers in HIV prevention and treatment have not substantially changed in Kenya and these data are still highly relevant due to the significant MTCT persisting in this setting.

## 5 CONCLUSIONS

Kenya, similar to other countries, is in need of innovative approaches to overcome challenges associated with optimal engagement with lifelong ART and HIV services among PWLWH. This study suggests that a cMM strategy may play an important role in the prevention of perinatal transmission of HIV as well as maternal and child health, and may also have positive effects on the cMMs themselves.

## COMPETING INTERESTS

The authors declare that they have no competing interests.

## AUTHORS’ CONTRIBUTIONS

AH, MO, LA, TO and JT developed the study design and protocol. SK carried out the literature search and assisted with the fine‐coding of qualitative data. GO conducted qualitative interviews. TO supported the implementation of both parts of the study. KO and KH provided support with the methodology and data analysis. All authors contributed to the thematic analysis and drafting of the findings. All authors have read and approved the final manuscript.

## FUNDING

The MOTIVATE study is supported by Award Number R01HD080477 from the US NIH/National Institute of Child Health and Human Development (Clinicaltrials.gov Protocol Record 14–0331). The content is solely the responsibility of the authors and does not necessarily represent the official views of the National Institute of Child Health and Human Development or the National Institutes of Health. Additional funding was provided by the University of Alabama at Birmingham (UAB) Sparkman Center for Global Health, UAB Graduate School, and UAB Center for AIDS Research.

## Data Availability

The data that support the findings of this study are available from the corresponding author (AH) upon reasonable request.
