# Supplementary material for: Experiences, perceptions and potential impact of community‐based mentor mothers supporting pregnant and postpartum women with HIV in Kenya: a mixed‐methods study
Source: J Int AIDS Soc. 2021 Nov 19;24(11):e25843. doi: 10.1002/jia2.25843 (PMC8604379; doi:10.1002/jia2.25843)
Supplement: Supplementary file 1 [file JIA2-24-e25843-s001.docx]

**COREQ – Consolidated criteria for reporting qualitative research - Checklist**

Tong A. Sainsbury P. Craig J. Consolidated criteria for reporting qualitative research (COREQ): a 32-item checklist for interviews and focus groups. International Journal for Quality in Health Care. 19(6):349-357

| **Item** | **Response** | **Location in manuscript (section, page number)** |
| --- | --- | --- |
| ***Domain 1: Research team and reflexivity*** | | |
| *Personal Characteristics* | | |
| 1. Interviewer/ facilitator | Experienced interviewers/moderators | Methods |
| 2. Credentials (in order of authorship) | AH holds DrPH, MA, MBA, MPH; MO holds MBChB, MSc, and PhD; LA is MD; KH holds PhD, KO holds MSc and completing DrPH; SK has BS; GO holds MPH; TO has BScN; TO has MBChB, MPH and PhD; JT PhD and MPH | Title Page |
| 3. Occupation | AH is Global Health outcomes researcher; MO is Global Health research scientist and medical doctor; LA is an Associate Professor and medical doctor; KH is an Assistant Professor; KO is biostatistician; SK is a public health practitioner; GO and TP are Program Coordinators; TO is a research scientist and medical doctor, JT is a Professor | Title Page |
| 4. Gender | AH, MO, LA, KH, SK, GO, JT identify as women; KO, TO, and TO identify as men | N/A |
| 5. Experience and training | All authors are technically and methodologically experienced researchers in Kenyan setting; interviewers/moderators were additionally trained by JT | Methods, Title Page |
| *Relationship with participants* | | |
| 6. Relationship established | Patients had prior relationship with healthcare workers from health facilities via utilized health services. | Methods |
| 7. Participant knowledge of the interviewer | Ethical approvals have been granted. Participants were informed about the reasons for the research via the invitation to participate and the consent form. | Methods |
| 8. Interviewer characteristics | No interviewer-related biases identified. | Competing Interests |
| ***Domain 2: Study design*** | | |
| *Theoretical framework* | | |
| 9. Methodological orientation and theory | Thematic analysis approach. The coding framework based on literature, topics from interview guides, and emerging themes from transcripts. | Methods |
| *Participant Selection* | | |
| 10.Sampling | Participants were purposively selected to include 24 community mentor mothers associated with the MOTIVATE parent trial. | Methods |
| 11. Method of approach | Participants were contacted in person by the research coordinator and asked to participate in an in-depth interview. | Methods |
| 12. Sample size | 24 community mentor mothers participated in in-depth interviews. | Methods |
| 13. Non-participation | All eligible and approached individuals agreed to participate in the study. | N/A |
| *Setting* | | |
| 14. Setting of data collection | In-depth interviews were conducted in private setting at their health care facilities. | Methods |
| 15. Presence of non-participants | No | N/A |
| 16. Description of sample | All participants were 18 years or older and provided their consent to participate in the study. | Methods, Ethical Approvals |
| *Data collection* | | |
| 17. Interview guide | A qualitative in-depth interview guide was developed based on a review of the literature, prior studies on pregnancy and HIV in this setting, and preliminary results from the formative phase of the parent MOTIVATE study. The main topics explored included: 1) experience working as a cMM (responsibilities, benefits/challenges, perceived impact of their work), 2) acceptability of cMMs in the community, 3) impact of this work on cMMs themselves, and 4) suggestions for improving the cMM intervention. Interview guide was translated to local languages (Kiswahili and Luo), and was also reviewed and approved by appropriate ethics review boards. | Methods |
| 18. Repeat interviews | N/A | N/A |
| 19. Audio/ visual recording | Interviews were digitally recorded, translated into English if applicable, and transcribed verbatim by professional transcriptionists, excluding any identifying information. All files were password-protected and stored in a secure location. | Methods |
| 20. Field notes | Interviewers took notes for purposes of assistance with transcription. | Methods |
| 21. Duration | Each interview lasted approximately 1 -1.5 hours. | Methods |
| 22. Data saturation | Data saturation was discussed and it was concluded that the data saturation was reached. | Methods |
| 23. Transcripts returned | N/A | N/A |
| ***Domain 3: Analysis and findings*** | | |
| *Data analysis* | | |
| 24. Number of data coders | Transcripts were coded by two researchers (AH and SK) using the Dedoose qualitative software, and consistency of coding between two individuals was established by initially double-coding the same transcripts and, after stablishing good agreement on codes and meanings, through frequent discussion and validation between coders and the wider team of investigators. The qualitative report and final themes were reviewed by all authors. | Methods |
| 25. Description of coding tree | Thematic analysis approach was utilized. The coding framework based on literature, topics from interview guides, and emerging themes from transcripts. | Methods |
| 26. Deviation of themes | Coding and analysis followed a thematic analysis approach. The coding framework was developed based on the literature, topics from interview guides, and emerging themes from transcripts. Summary of themes and key results are provided in the Results section. | Methods, Results |
| 27. Software | Dedoose qualitative software program (Sociocultural Research Consultants, LLC) was utilized. | Methods |
| 28. Participant checking | Findings were disseminated at participating health facilities and communities. | N/A |
| *Reporting* |  |  |
| 29. Quotations presented | Participant quotations are provided to illustrate the themes and attributed to participants. De-identified participant characteristics are provided for each quote to distinguished between participant’s age and number of living children. | Results |
| 30. Data and findings consistent | Yes in qualitative part, some divergence in the integrated findings. | Results, Discussion |
| 31. Clarity of major themes | Five major themes are clearly presented and summarized in Results. | Results |
| 32. Clarity of minor themes | Minor themes were not discussed to keep focus. | N/A |
